# Metagenomic next-generation sequencing: a game-changer in the diagnosis of unique intraocular infections

**DOI:** 10.1038/s41433-025-04073-w

**Published:** 2025-10-25

**Authors:** Zohar Habot-Wilner, Michael Ostrovsky, Dinah Zur, Shulamit Schwartz, David Hagin, Avi Gadoth, Ronen Ben-Ami, Yael Paran, Hanoch Goldshmidt, Matan Slutzkin, Amos Adler, Katya Levytskyi

**Affiliations:** 1 Division of Ophthalmology, Tel Aviv Sourasky Medical Center, Tel Aviv, Israel; 2 Gray Faculty of Medical and Health Sciences, Tel Aviv University, Tel Aviv, Israel; 3 Department of Ophthalmology, Kaplan Medical Center, Faculty of Medicine, Hebrew University of Jerusalem, Jerusalem, Israel; 4 Allergy and Clinical Immunology Unit, Tel Aviv Sourasky Medical Center, Tel Aviv, Israel; 5 Neurology Division, Tel Aviv Sourasky Medical Center, Tel Aviv, Israel; 6 Infectious Disease Unit, Tel Aviv Sourasky Medical Center, Tel Aviv, Israel; 7 Division of Laboratories, Tel Aviv Sourasky Medical Center, Tel Aviv, Israel; 8 The Mina & Everard Goodman Faculty of Life Sciences, Bar-Ilan University, Ramat-Gan, Israel; 9 Microbiology Laboratory, Tel Aviv Sourasky Medical Center, Tel Aviv, Israel

**Keywords:** Uveal diseases, Vision disorders

## Abstract

**Objective:**

To thoroughly describe unique intraocular infections diagnosed by metagenomic next-generation sequencing (mNGS).

**Methods:**

A retrospective case series of patients presenting with challenging atypical intraocular infections at Tel Aviv Sourasky Medical Center during 2024. Clinical and demographic data, as well as mNGS results were extracted from patient records. mNGS was performed on the Illumina NextSeq500 platform using a custom bioinformatics pipeline. The following parameters were examined: Reads Per Million, Reads Per Million-ratio to negative control and E-index (K-mers*coverage/reads).

**Results:**

The study included three patients with novel presentations of intraocular infections, manifesting with atypical clinical manifestations and negative routine diagnostic workups. mNGS allowed the identification of *Cytomegalovirus* in a 43-year-old male with a history of autosomal dominant hyper-IgE syndrome, *Bartonella henselae* infection manifesting with photoreceptoritis, retinal vasculitis and global retinal dysfunction in a healthy 28-year-old female, and polymicrobial endophthalmitis with *Rothia mucilaginosa* and *Pantoea agglomerans* following intravitreal faricimab injection for neovascular age-related macular degeneration in an 81-year-old male. Treatment regimens were adjusted based on mNGS results.

**Conclusions:**

Metagenomic next-generation sequencing has an important role in the diagnosis of challenging intraocular infections. It enables comprehensive pathogen identification and enhances the precision of treatment strategies.

## Introduction

Intraocular infections (IOI) are sight-threatening conditions that require prompt diagnosis and treatment. Their diagnosis is challenging due to clinical overlap with non-infectious inflammatory disorders, and difficulty in identifying diverse aetiologies using traditional laboratory methods. IOI can be caused by bacteria, viruses, fungi, or parasites, and their prevalence varies widely by geographic region, host factors, and environmental conditions [1, 2]. Despite the availability of diagnostic tools, the causative pathogen remains unidentified in many presumed infectious cases, highlighting the need for more robust diagnostic methods [3].

Microbiological cultures allow microorganism detection without prior clinical clues. However, they are hindered by lengthy processing times and may miss fastidious or slow-growing pathogens, resulting in relatively low sensitivity ranging between 40% and 70% [4, 5]. Polymerase chain reaction (PCR) is well suited for identifying suspected pathogens from relatively large ocular fluid samples, offering more rapid and reliable results than culture. However, its reliance on predefined primers introduces the risk of false negatives if the pathogen’s genome is not well-matched, and false positives due to cross-reactivity [6–8].

Metagenomic next-generation sequencing (mNGS) represents a novel technique in the diagnosis of IOI. It enables unbiased, comprehensive analysis of all genetic material in a clinical sample, allowing rapid simultaneous detection of all potential ocular pathogens from a small sample obtained from the anterior chamber (AC) or the vitreous [9–11]. As all genomic material in the sample is sequenced, this technique is especially valuable when the pathogen is fastidious or unidentified through conventional methods.

Initial studies reporting on the use of mNGS for the detection of IOI emerged from the United States [9, 10, 12], followed by reports from research groups across Asia, North America, and Europe, as demonstrated in Supplementary Table 1. While these studies reported a diverse spectrum of intraocular pathogens, they did not thoroughly describe the clinical cases. In this study, we present unique cases of intraocular infections diagnosed by the mNGS technique.

## Methods

This retrospective study was conducted at Tel Aviv Sourasky Medical Centre, Tel Aviv, Israel during 2024. The study was approved by the local Institutional Review Board (TLV-0730-23) and adhered to the Declaration of Helsinki.

The electronic medical records were reviewed to extract demographic and clinical data, including: sex, age at presentation, ethnic origin, systemic comorbidities, laterality of eye disease, best-corrected visual acuity, intraocular pressure (IOP), ocular findings, ocular and systemic workup and treatments. Uveitis was classified according to the Standardization of Uveitis Nomenclature (SUN) Working Group classification [13].

### Metagenomic next-generation sequencing

Ocular samples, from the AC and/or vitreous, were filtered for human nucleic acid (NA) depletion using the Devin Microbial DNA Enrichment Kit (Micronbrane Medical) following the manufacturer’s protocol. After filtration, processed samples were stored at −20 °C. NA extraction was performed using the same kit with the addition of a negative control of RNAse/DNAse free molecular grade water undergoing the same processing steps from this point. Internal control using ZymoBIOMICS Spike-in Control I (Zymo; High Microbial Load) was added to each sample including the negative control to evaluate information loss in downstream analysis. Extracted NA quality was assessed with a NanoDrop spectrophotometer, and DNA density was measured using the Qubit dsDNA HS Assay Kit (Thermo Fisher Scientific). Library preparation was conducted using the Unison Ultralow DNA NGS Library Preparation Kit (Micronbrane Medical). Size selection and cleanup of the prepared libraries were performed using Ampure XP beads with a two-step process targeting fragment lengths of between 200–500 base pairs. Each library was individually quantified using the Qubit dsDNA HS Assay Kit and the fragment size distribution was determined using the High Sensitivity DNA Kit (Agilent) on an Agilent 2100 Bioanalyzer. Equimolar and equal volume of DNA libraries were combined. The pooled library was loaded onto an Illumina NextSeq 500 sequencer for a paired-end 150-cycle run and sequenced to generate a minimum of 5 million reads per sample.

Bioinformatics Analysis Sequencing data were analysed using an in-house Snakemake-based computational pipeline. Raw paired-end FASTQ reads underwent initial quality assessment with FastQC (v0.11.9). Adapter trimming and quality filtering were then performed using Trimmomatic (v0.39). To eliminate host-derived sequences, the trimmed reads were aligned to the human reference genome (GRCh38) using Bowtie2 (v2.5.1). Reads mapping to the human genome were discarded and a secondary filtering step using SNAP (v2.66.1) was employed to ensure the removal of any residual human contamination. Remaining reads were mapped against the internal-control sequences (Imtechella halotolerans and Allobacillus halotolerans) to count and remove them from further classification. Surviving reads underwent taxonomic classification using KrakenUniq (v1.0.4), which provided unique read counts for each taxonomic group. To normalise for variations in sequencing depth, the Reads Per Million (RPM) metric was calculated. To mitigate random noise in classification generated from mapping small fragments against a large database and from potential sample processing contaminations, each RPM assigned to a taxon was divided by the corresponding taxon RPM in the negative control (a value of 1 was used if the taxon did not appear in the negative control classification). This metric is designated as RPM-ratio calculated using custom Python scripts. The parameter used to determine the validity of the results was the RPM-r with a threshold of >10.

## Results

### Case 1

**Fig. 1:**
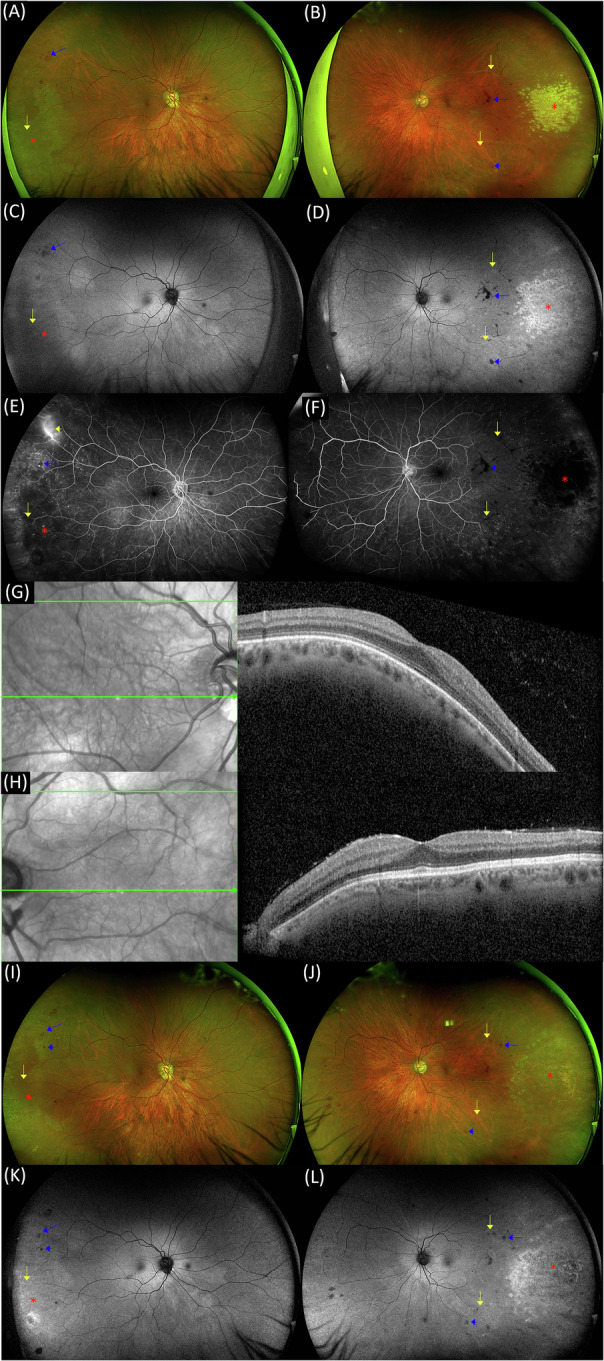
Multimodal imaging of bilateral cytomegalovirus retinitis and retinal vasculopathy in STAT3-associated hyper-IgE syndrome. **A**, **B** Ultrawide-field fundus photos at presentation, demonstrating areas of retinitis as temporal whitish lesions (red asterisk), retinal haemorrhages (blue arrow), white-centred retinal haemorrhages (blue arrowhead), and occluded blood vessels (yellow arrows). **C**, **D** Ultrawide-field FAF at presentation, demonstrating the whitish areas as hyper-autofluorescence (red asterisk), the retinal haemorrhages as hypo-autofluorescence (blue arrow), white-centred retinal haemorrhages as hypo- and hyper-autofluorescence (blue arrowhead), and occluded blood vessels (yellow arrows). **E**, **F** FA demonstrating multiple small peripheral hyperfluorescent foci, blockage by retinal haemorrhages (blue arrow) and by the temporal retinal lesion (red asterisk), and occluded blood vessels (yellow arrows). The RE had leakage from a peripheral superotemporal blood vessel (yellow arrowhead). **G**, **H** Macular OCT demonstrating a myopic structure and vitreal hyperreflective foci. **I**, **J**, **K**, **L** Fundus photos and FAF at three months post-presentation, demonstrating resolution of retinitis (red asterisk), retinal haemorrhages (blue arrow), white-centred retinal haemorrhages (blue arrowhead), and occluded blood vessels (yellow arrows).

A 43-year-old man with autosomal dominant (AD) signal transducer and activator of transcription 3 (STAT3) hyper-IgE syndrome (HIES) presented with a two-day history of fever (40 °C) and tremors. He underwent mechanical aortic valve replacement due to fulminant *Staphylococcus aureus* infective endocarditis (IE) four years prior, with prophylactic antibiotic treatment since, including trimethoprim/sulfamethoxazole (800/160 mg x2/day), azithromycin (250 mg x3/week), and monthly immunoglobulin replacement therapy ( ~ 500 mg/Kg/month). The patient was admitted to rule out mechanical valve endocarditis and referred for ocular examination. His right eye (RE) had mild amblyopia. Best-corrected visual acuity (BCVA) was 6/12 in the RE and 6/7.5 in the left eye (LE), and IOP was 12 mmHg in both eyes (BE). The RE had a normal anterior segment, clear lens, +0.5 vitreous haze, the optic disc was tilted with a peripapillary atrophy (PPA) and the macula was normal. There was a whitish area in the temporal retina, with several occluded peripheral veins and a few retinal haemorrhages within and superior to this area (Fig. 1A). The LE had mild conjunctival injection, small scattered keratic precipitates (KPs), +3 cells, clear lens, +0.5 vitreous haze. The optic disc was tilted with a PPA. There were multiple whitish retinal lesions, some confluent, in the temporal retina, and retinal haemorrhages involving the macula and surrounding the lesions. In addition, there were few extra-macular white-centred retinal haemorrhages and vein and artery occlusions (Fig. 1B). Fundus autofluorescence (FAF) demonstrated in BE the whitish areas as hyper-autofluorescence, the retinal haemorrhages as hypo-autofluorescence and emphasised the occluded vessels (Fig. 1C, D). Fluorescein angiography (FA) demonstrated in BE multiple small peripheral hyperfluorescent foci, in addition to blockage by retinal haemorrhages and by the temporal retinal lesion. The RE had leakage from a peripheral superotemporal blood vessel and an occluded temporal peripheral vein, and the LE had temporal vein and artery occlusions (Fig. 1E, F). Macular optical coherence tomography (OCT) demonstrated in BE a myopic structure, and vitreal hyperreflective foci (Fig. 1G, H). Extensive work-up, including blood cultures, transthoracic and transoesophageal echocardiography (TTE), and cardiac CT showed no evidence of intracardiac infection. Due to the unexpected ocular findings and known primary immunodeficiency disorder, a LE AC tap was performed and a sample was sent for mNGS analysis. Surprisingly, mNGS was strongly positive for cytomegalovirus (CMV)(Table 1). To validate this unexpected result, PCR for CMV was performed and found positive. The patient was diagnosed with bilateral CMV retinitis and retinal vasculopathy manifesting with retinal blood vessel occlusions and retinal haemorrhages. Peripheral blood PCR was negative for CMV. He was treated with oral valganciclovir (900 mg x2/day) for 3 weeks, followed by 450 mg x2/day, four-weekly intravitreal (IVT) 2 mg/0.05 mL ganciclovir injections to the LE, hourly dexamethasone sodium phosphate (Sterodex^®^) while awake, nightly dexamethasone ointment (Maxitrol^®^), and tropicamide (Mydramide^®^) x3/day. At last follow-up, 3 months after his initial presentation, the patient was on valganciclovir (450 mg x2/day) treatment. His BCVA was 6/12 in the RE and 6/6 in the LE, and IOP was 10 mmHg bilaterally. CMV retinitis regressed under treatment. There were retinal haemorrhages and peripheral occluded blood vessels secondary to vasculopathy (Fig. 1I–L).

**Table 1:** Metagenomic next-generation sequencing results.

Case number	Detected pathogen	cov(0–1)	RPM	RPM-r	E score
Case #1	Cytomegalovirus	0.563	9028.83	9028.83	0.6
Case #2	Bartonella henselae	0.01329	5696.29	1.27	0.023
Case #3	Rothia mucilaginosa	0.000268	15.22	15.22	0.0008
	Pantoea agglomerans	0.00005	12.46	12.46	0.00013

E score = (unique k-mers/read count per taxon)*coverage.

cov; coverage, RPM; reads per million, RPM-r; reads per million-ratio.

### Case 2

**Fig. 2:**
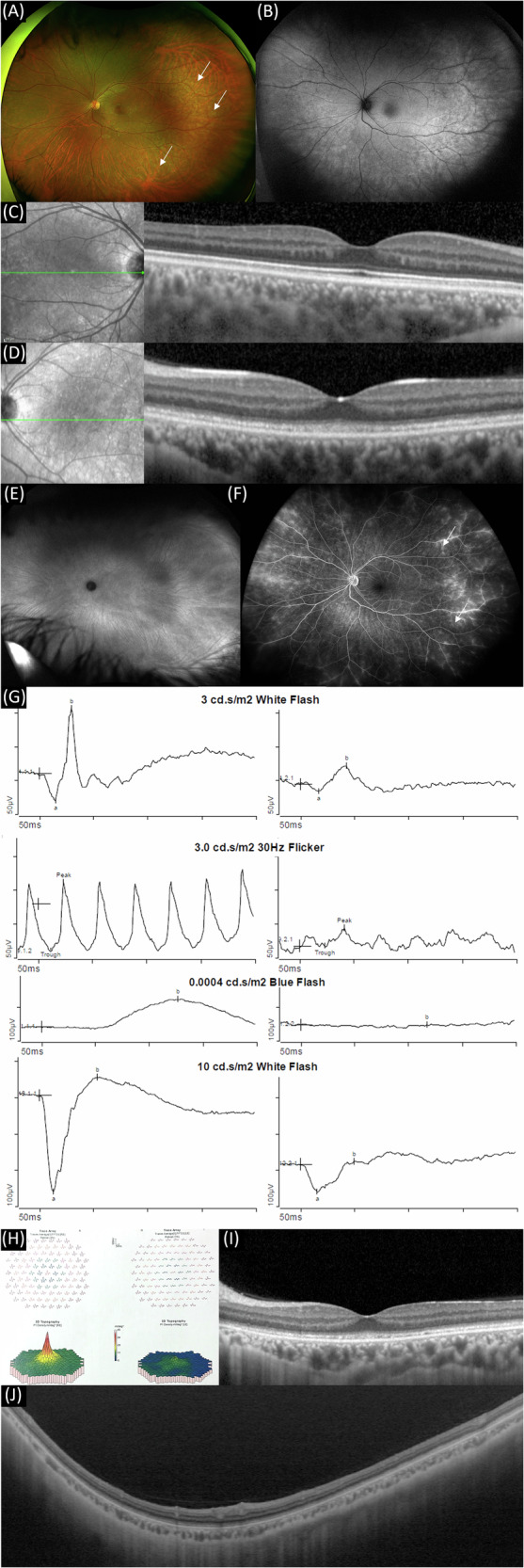
Multimodal imaging and electrophysiologic findings in Bartonella henselae–associated photoreceptoritis and retinal vasculitis. Initial presentation (**A**) ultrawide-field fundus photo of the left eye, showing a normal optic disc and mid-peripheral hypopigmentary changes (white arrows). **B** Ultrawide-field FAF of the left eye showing hyperautofluorescence in the mid-periphery. **C** Normal macular OCT of the right eye. **D** Macular OCT of the left eye, showing fuzziness and granularity of the interdigitation and ellipsoid zone. **E** Ultrawide-field late-phase ICGA of the left eye within normal limits, ruling out choroidal involvement. **F** Ultrawide-field mid-phase FA of the left eye showing staining with mild leakage from peripheral temporal veins and mild leakage from retinal capillaries. **G** Full-field ERG showing normal response in the RE, and moderately reduced cone response in the LE. The rod response to 0.001 cd was absent, and the mixed scotopic response was markedly reduced with marginal electronegative waveforms. **H** Multifocal ERG showing normal foveal and macular mal in the right eye and absent foveal peak with reduced responses in the whole tested area, indicating diffuse functional macular cone impairment. **I** Macular OCT of the left eye, showing fuzziness and granularity of the interdigitation and ellipsoid zone. **J** Widefield OCT of the inferior retina in LE showing fuzziness and granularity of the interdigitation and ellipsoid zone.

A 28-year-old female with a history of bariatric surgery 3 years before presentation with weight loss of 50 kg, presented with a six-month history of decreased vision in her LE. She denied any prior ocular history, trauma, or systemic symptoms such as fever, lymphadenopathy, or malaise. The patient reported contact with a home pet cat. Post-surgical vitamin levels and routine chemistry panels were within normal limits. On examination, her visual acuity was 6/6 in the RE and 6/20 in the LE. IOP was 12 mmHg in BE. The RE was normal. LE examination revealed a normal anterior segment, clear vitreous and lens, and a normal optic disc. However, the macular reflex was dull, with mid-peripheral hypopigmented areas (Fig. 2A). Visual field testing 10-2 was normal in the RE, whereas the LE showed decreased sensitivity (mean deviation −4.3). Macular OCT, FAF and FA of the RE were normal. FAF of the LE revealed hyperautofluorescence predominantly in the mid-peripheral retina (Fig. 2B). OCT of the LE demonstrated diffuse disruption of the hyperreflective bands of the ellipsoid zone (EZ) and the interdigitation zone (IZ) that correspond to the photoreceptor layers (Fig. 2C, D). Indocyanine green angiography (ICGA) was unremarkable in BE (Fig. 2E). FA showed staining with mild leakage from peripheral temporal veins and diffuse staining from retinal capillaries (Fig. 2F). Full-field electroretinography (ERG) was normal in the RE but demonstrated moderately decreased peripheral cone response and absent rod responses in the LE (Fig. 2G). Multifocal ERG was normal in the RE, but showed diffusely reduced macular cone responses in the LE, including the foveal response (Fig. 2H). A comprehensive systemic workup was performed. Laboratory testing, including CBC, liver and kidney functions, vitamin A, B1, B6, and B12 levels, syphilis serology (*Treponema pallidum* haemagglutination; TPHA), and HIV was unremarkable. Chest X-ray was normal. Initial serological testing for *Bartonella*, including IgM and IgG antibodies, was negative, as were repeat tests 3 months after initial presentation. The patient was diagnosed with non-infectious diffuse photoreceptoritis and retinal vasculitis. Brain MRI was performed to rule out intracranial vasculitis and was normal. She was started on 40 mg oral prednisone (1 mg/kg). After two weeks of treatment, VA and macular OCT findings remained unchanged. A LE AC tap was performed, and a sample was sent for mNGS analysis, which revealed *Bartonella henselae* (Table 1). This unexpected result prompted validation via PCR testing, which also yielded a positive result. The patient was diagnosed with LE Bartonella infection, causing a significant ocular immune reaction, manifesting with photoreceptoritis, retinal vasculitis and global retinal dysfunction. Treatment with oral doxycycline (100 mg x2/day) and rifampin (300 mg x2/day) was initiated, and oral corticosteroids were tapered. After two months of antibiotic therapy, LE VA improved to 6/15. However, at 5 months post-presentation, while on low-dose prednisone (5 mg/day), she experienced worsening of her vision to 6/20. There was no change in clinical findings, including OCT, FAF, and FA imaging (Fig. 2I, J). This prompted treatment with IV methylprednisolone (1 gr/day for 3 days) to address the inflammatory component, and the patient was further treated with oral prednisone (40 mg/day) which was gradually tapered down until cessation. At last visit, 7 months from first presentation, VA remained 6/20, and OCT, FAF, and ERG findings remained unchanged.

### Case 3

An 81-year-old man with diabetes mellitus type 2 presented with a two-week history of visual acuity deterioration, persistent redness, and discomfort in the LE. His ocular history included LE neovascular age-related macular degeneration (nvAMD) for which he had been receiving IVT faricimab injections. The last injection was given four days before his eye symptoms began. On examination, BCVA was 6/9 in the RE and counting fingers (CF) in the LE. IOP was 10 mmHg in BE. RE examination revealed a normal anterior segment, posterior chamber intraocular lens (PCIOL), a clear vitreous, the optic disc was normal with extensive PPA and retinal pigment epithelium (RPE) changes superior to the optic disc, the fovea was spared and the peripheral retina was normal. LE examination revealed conjunctival irritation, corneal stromal haze with Descemet folds, inferotemporal KPs, the AC was deep with +4 cells, +4 flare and a 1 mm hypopyon, PCIOL, +4 vitreous haze with no ability to appreciate the fundus. Ultrasound examination of the LE demonstrated a significant vitreous opacity and an attached retina. The patient was diagnosed with LE endophthalmitis and underwent pars plana vitrectomy (PPV) with IVT 1 mg/0.1 mL vancomycin and 2.25 mg/0.1 mL ceftazidime injection. A smear from the vitreous fluid sample showed many leucocytes, with no evidence of bacteria or fungi, and no growth was found on microbial cultures. A sample was also sent for mNGS analysis. The patient was treated with hourly vancomycin and ceftazidime eyedrops, which were titrated over three weeks. In addition, dexamethasone sodium phosphate (Sterodex^®^) x4/day eyedrops, nightly dexamethasone ointment (Maxitrol^®^), and tropicamide (Mydramide^®^) x3/day eyedrops were added. mNGS revealed a polymicrobial infection with *Rothia mucilaginosa* and *Pantoea agglomerans* (Table 1). Topical treatments were tapered down. At three months after his initial presentation and 3 weeks post treatment cessation, the patient’s BCVA was 6/8.5 in the RE and 6/40 in LE, and IOP was 13 mmHg in BE. RE examination remained unchanged. LE examination revealed normal conjunctiva and cornea, a deep and clear AC, PCIOL, clear vitreous, the optic disc had a 0.3 cup-to-disc ratio, with PPA. There was a sub-foveal scar surrounded by RPE changes and few small drusen and the retina was attached.

## Discussion

This study describes the application of mNGS in challenging intraocular infections, emphasising the crucial role of this novel diagnostic modality. mNGS offers a capacity for broad-spectrum, single-test pathogen detection, allowing clinicians to accurately identify both common and atypical pathogens, even in cases where conventional methods fail [9–11].

The first case describes a 43-year-old man with autosomal dominant STAT3 HIES. This is a multi-organ syndrome classically characterised by a triad of elevated serum IgE, eczema, and recurrent skin and respiratory tract infections. STAT3-HIES is also associated with non-immune features, including vascular abnormalities, facial dysmorphism and skeletal abnormalities [14]. Typically, patients also show susceptibility to *S. aureus* and *Candida spp* infections, while viral infections are rarely reported. The patient presented with two unique ocular manifestations, CMV retinitis and retinal vasculopathy. AD-HIES is caused by mutation in *STAT3*, which is required for the generation and development of IL17-producing CD4 + T-cells [15]. Therefore, AD-HIES patients are typically susceptible to a narrow spectrum of pathogens, including *Staphylococcus aureus* and *Candida albicans*. In addition, STAT3 also plays a role in CD8 development through IL21 signalling, and higher incidence of VZV reactivation and EBV viremia was suggested [16, 17]. This unique case is the first to report CMV retinitis and the additional described ocular findings in an AD-HIES patient. The definitive diagnosis of CMV retinitis was done by mNGS. Since CMV retinitis was not yet described in AD-HIES patients, we validated the result using CMV-specific PCR. The patient was successfully treated with a combination of systemic valganciclovir and IVT ganciclovir injections resulting in resolution of the infection.

The second case describes a young woman with Bartonella infection causing significant ocular immune reaction, with photoreceptoritis, retinal vasculitis, and global retinal dysfunction. ICGA was normal, indicating no involvement of the choriocapillaris, thus distinguishing primary photoreceptor damage from secondary causes. FAF showed hyperautofluorescence due to a window defect, caused by loss of photopigment in the outer segments of the photoreceptors with an intact RPE, and the OCT demonstrated the diffuse disruption of the EZ [18]. ERG revealed unspecific global reduction of retinal function, with dominant rod dysfunction, as well as impaired macular and peripheral cone dysfunction. The mixed response showed a marginal electronegative pattern, suggesting significant immune retinopathy. This is the first documented case of ocular *Bartonella henselae* infection manifesting as primary photoreceptoritis and immune retinopathy. Ocular cat scratch disease (CSD) may present with various clinical manifestations, most commonly neuroretinitis [19, 20]. The diagnosis of ocular bartonellosis is challenging, as it is difficult to grow *B. henselae* in culture. In addition, serologic testing (immunofluorescence or enzyme immunoassay) has variable sensitivity and specificity. Seroconversion may occur within weeks, making serology time-sensitive, and diagnosis is often based on the presence of IgG in the absence of IgM, which is also not easily detected. PCR can detect different *Bartonella* species, however, its sensitivity is lower than that of serology. PCR from pus aspirated from lymph nodes or the primary inoculation site is highly sensitive and specific and is particularly useful for rapid definitive diagnosis in seronegative patients [21, 22]. Although our patient was a cat owner, she did not exhibit any systemic signs of CSD, including lymphadenopathy. Thus, we did not consider blood or lymph node PCR following the negative serology result. mNGS enabled the unique diagnosis of this case, which was afterward validated by AC aqueous humour PCR. The patient was treated with a combined regimen of antibiotics and corticosteroids. However, the condition remained mainly unchanged, with persistent morphological and functional photoreceptor impairment. The therapeutic challenge in this case stemmed from the photoreceptor involvement in the inflammatory process, which may result in irreversible damage, particularly in cases of delayed diagnosis. Unfortunately, the patient did not respond despite Bartonella-directed antibiotics and high-dose corticosteroid therapy.

The third case represents a rare case of polymicrobial endophthalmitis following intravitreal faricimab injection, involving two uncommon pathogens: *Rothia mucilaginosa* and *Pantoea agglomerans*. Polymicrobial endophthalmitis is an exceedingly rare phenomenon, reported following ocular trauma or post-operatively. To date, only a few cases of polymicrobial endophthalmitis following an IVT injection were reported [23, 24]. *Rothia mucilaginosa* is a Gram-positive, coagulase-negative encapsulated coccus within the family *Micrococcaceae*. Although typically part of the normal flora of the human oral cavity, upper respiratory tract, and gastrointestinal tract, it has also been implicated in ocular infections following cataract surgery [25], iStent inject implantation [26], and late-onset bleb-associated endophthalmitis [27]. *Pantoea agglomerans*, is a Gram-negative, aerobic bacillus in the family Enterobacteriaceae, commonly found in feculent material, plants, and soil. It is a known cause of infections following penetrating trauma involving vegetative material and catheter-related bacteraemia [28]. *Pantoea agglomerans* has been isolated in several cases of post-traumatic and post-cataract surgery endophthalmitis [29–32], as well as two cases of endogenous endophthalmitis [33, 34]. It is of utmost importance to accurately identify the causative pathogens in polymicrobial endophthalmitis to tailor the appropriate treatment. However, cultures were previously reported to only identify between a third and two-thirds of pathogens causing endophthalmitis [4, 5]. This may be due to small sample volume, low bacterial load, prior antibiotic use, or the pathogens’ fastidious nature. In addition, mixed PCR amplicons of polymicrobial infection cannot be sequenced without cloning or NGS methodologies. In our patient, the vitreous culture was found negative, and the mNGS enabled the unexpected diagnosis of polymicrobial infection with two uncommon pathogens. Multiplex PCR and recent developments in Microarray analysis allow rapid, cost-effective detection of a relatively large number ( ~ 20–30) of organisms within a single sample [35]. However, these tests are typically designed for the detection of the most common pathogens for relatively common clinical syndromes (e.g., microbial keratitis) and thus cannot detect relatively rare pathogens in less common clinical syndromes as described here. This is the first reported case of polymicrobial endophthalmitis post-intravitreal faricimab injection. The patient was managed with a suitable antibiotic regimen, with complete resolution of the infection and visual acuity improvement.

This study’s limitations include its retrospective nature and small sample size. However, our aim was not to introduce the mNGS technique in a large cohort, but rather to emphasise its extraordinary value in the diagnosis of rare challenging IOI. It should be noted that the reliance on mNGS requires advanced laboratory infrastructure and bioinformatics and that our study, similarly to other studies on mNGS, represents the use of a specific bioinformatics pipeline developed at our medical centre.

In conclusion, this case series highlights the crucial role of mNGS in the diagnosis of atypical challenging intraocular infections. Through the application of mNGS, we were able to accurately identify a diverse array of pathogens, reveal rare cases that were not described before, and expand our understanding of intraocular infections. The use of mNGS allowed us to achieve the appropriate therapeutic regimens, leading to favourable clinical outcomes in most cases. mNGS represents a powerful diagnostic tool in cases of intraocular infection where standard microbiological and serologic assays fail to identify the causative pathogen. Future studies should determine the impact of mNGS on clinical outcomes in larger patient cohorts.

## Summary

### What was known before:

- Intraocular infections are often challenging to diagnose due to overlapping clinical features and limitations of culture- and polymerase chain reaction-based methods.
- Metagenomic next-generation sequencing (mNGS) enables comprehensive, unbiased pathogen detection which could improve ophthalmic diagnostic practice.

### What this study adds:

- This study reports new data on unique, previously unreported cases of intraocular infections identified by the mNGS technique.
- New infectious ocular manifestations are described: Cytomegalovirus retinitis along with retinal vasculopathy in autosomal dominant hyper-IgE syndrome, Cat scratch disease manifesting as photoreceptoritis, retinal vasculitis and global retinal dysfunction and polymicrobial endophthalmitis with Rothia mucilaginosa and Pantoea agglomerans following faricimab injection.

## Supplementary information

Supplemental Table 1

Reporting Checklist

## Data Availability

The datasets generated during and/or analysed during the current study are available from the corresponding author on reasonable request.
